# Xylans of Red and Green Algae: What Is Known about Their Structures and How They Are Synthesised?

**DOI:** 10.3390/polym11020354

**Published:** 2019-02-18

**Authors:** Yves S.Y. Hsieh, Philip J. Harris

**Affiliations:** 1 Division of Glycoscience, Department of Chemistry, School of Engineering Sciences in Chemistry, Biotechnology and Health, Royal Institute of Technology (KTH), AlbaNova University Centre, SE-106 91 Stockholm, Sweden; 2 School of Biological Science, The University of Auckland, Private Bag 92019, Auckland, New Zealand

**Keywords:** cell walls, charophyte green algae, chlorophyte green algae, land plants (embryophytes), polysaccharides, rhodophytes, 1,3-β-d-xylans, 1,3, 1,4-β-d-xylans, 1,4-β-d-xylans

## Abstract

Xylans with a variety of structures have been characterised in green algae, including chlorophytes (Chlorophyta) and charophytes (in the Streptophyta), and red algae (Rhodophyta). Substituted 1,4-β-d-xylans, similar to those in land plants (embryophytes), occur in the cell wall matrix of advanced orders of charophyte green algae. Small proportions of 1,4-β-d-xylans have also been found in the cell walls of some chlorophyte green algae and red algae but have not been well characterised. 1,3-β-d-Xylans occur as triple helices in microfibrils in the cell walls of chlorophyte algae in the order Bryopsidales and of red algae in the order Bangiales. 1,3;1,4-β-d-Xylans occur in the cell wall matrix of red algae in the orders Palmariales and Nemaliales. In the angiosperm *Arabidopsis thaliana*, the gene *IRX10* encodes a xylan 1,4-β-d-xylosyltranferase (xylan synthase), and, when heterologously expressed, this protein catalysed the production of the backbone of 1,4-β-d-xylans. An orthologous gene from the charophyte green alga *Klebsormidium flaccidum*, when heterologously expressed, produced a similar protein that was also able to catalyse the production of the backbone of 1,4-β-d-xylans. Indeed, it is considered that land plant xylans evolved from xylans in ancestral charophyte green algae. However, nothing is known about the biosynthesis of the different xylans found in chlorophyte green algae and red algae. There is, thus, an urgent need to identify the genes and enzymes involved.

## 1. Introduction

After cellulose, xylans are the most abundant polysaccharides in the cell walls of land plants (embryophytes) [1]. In contrast to cellulose, which occurs in the form of microfibrils, xylans occur in the matrix phase of cell walls. Because of their occurrence in land plant cell walls, xylans are a major dietary component of foods for humans and ruminant animals, as well as having a variety of biotechnological applications. For example, biomass containing high proportions of cell walls rich in xylans, generated as agricultural and forestry wastes, has been used to produce biofuels, particularly bioethanol [2]. The xylans in corncobs and birch wood are also used in the manufacture of xylitol, which is used as a low-calorie sweetener that was first developed during World War II when sucrose was in short supply. Xylitol is made in large amounts from the xylose in biomass hydrolysates, with the conversion carried out mostly by yeast strains [3].

**Figure 1:**
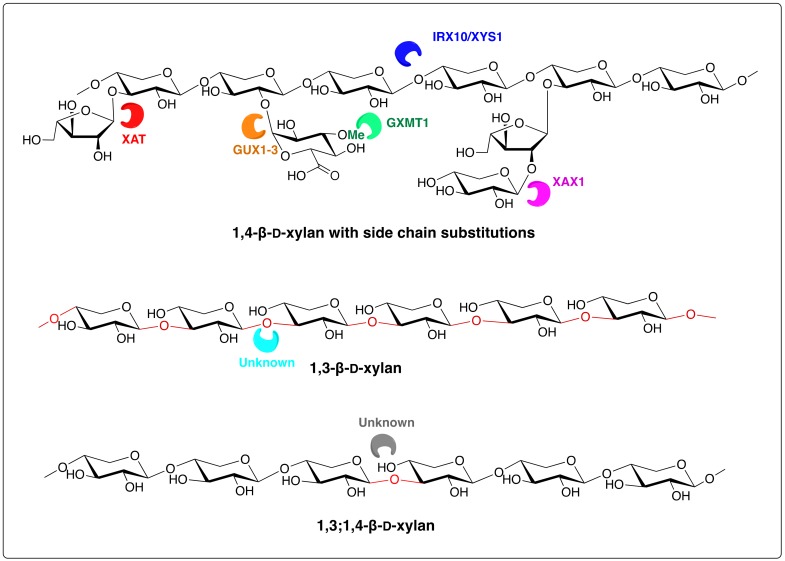
Structures of a 1,4-β-d-xylan with side-chain substitutions (a heteroxylan) as in the glucuronoarabinoxylans of the grass family (Poaceae) and the homoxylans 1,3-β-d-xylan and 1,3;1,4-β-d-d-xylan. The formation of the linkages shown are catalysed by the following enzymatic proteins: IRX10/XYS1 (irregular xylem 10/xylan synthase 1); XAT (xylan arabinosyl transferase); GUX1-3 (glucuronic acid substitution of xylan 1-3); GXMT1 (glucuronoxylan methyl transferase 1); and XAX1 (xylosyl arabinosyl substitution of xylan 1).

In land plants, there is considerable variation in the structures of the xylans depending on phylogenetic position and on whether the xylans are in primary or secondary cell walls [4,5,6,7,8]. Land plant xylans are composed of a linear backbone of 1,4-linked β-d-xylopyranosyl (β-d-Xyl*p*) residues, i.e., they are 1,4-β-d-xylans. However, it is unlikely that homoxylans occur in the cell walls of these plants [9]. Instead, they are heteroxylans with side chains containing various other monosaccharides, such as α-l-arabinofuranose (α-l-Ara*f*) and 4-*O*-methyl-α-d-glucuronic acid (MeGlc*p*A) or α-d-glucuronic acid (Glc*p*A), as well as oligosaccharides, acetyl groups and, in some taxa, phenolic acid esters, such as ferulate or *p*-coumarate esters. In the lignified cell walls of eudicotyledons, the side chains are usually MeGlc*p*A (or Glc*p*A) residues attached at *O*-2. However, some of the most complex xylans (glucuronoarabinoxylans, Figure 1) occur in the grasses (family Poaceae within the monocotyledons). In addition to Glc*p*A (or MeGlc*p*A), they have Ara*f* residues attached usually at *O*-3, and which may be esterified by ferulate, or less commonly by *p*-coumarate. Other, more complex side chains such as the disaccharide β-d-Xyl*p*-(1,2)-α-l-Ara*f*-(1,3)- also commonly occur (Figure 1). Further complexity occurs in the xylans in the secondary walls of eudicotyledons, many monocotyledons (but not those of the grasses), and gymnosperms, in the form of the oligosaccharide sequence 4)-β-d-Xyl*p*-(1,4)-β-d-Xyl*p*-(1,3)-α-l-Rha*p*-(1,2)-α-d-Gal*p*A -(1,4)-d-Xyl at the reducing end. The function of this is uncertain, but it is considered to be related in some way to xylan biosynthesis [8]. Heteroxylans occur even in the bryophytes, which make up the most basal lineages of the land plants, with the cell walls of the moss *Physcomitrella patens* containing xylans with Glc*p*A side chains (glucuronoxylans) [10]. 

In contrast to land plants, especially the angiosperms, much less is known about the xylans that occur in the cell walls of the green and red algae. Together, these two groups of algae contain some 15,000 species, and the cell wall polysaccharides have been examined in only a small proportion of these. Nevertheless, some of these species contain xylans with structures that are different from those found in the cell walls of land plants. In addition to the green and red algae, the kingdom Plantae (Archaeplastida) includes the glaucophytes (Glaucophyta) that are a small group of freshwater algae forming the most basal, branching lineage [11]. However, nothing is known about the occurrence of xylans in their cell walls. Outside of kingdom Plantae, the brown algae (class Phaeophyceae) is another large group of algae (1500–2000 species) that produces considerable amounts of biomass. They form part of the kingdom Chromista and, as far as we are aware, there are no reports of xylans occurring in their cell walls [12,13]. In this review, we provide a brief overview of the structural variation in the xylans found in the cell walls of green and red algae. We also briefly discuss what is known about the genes and cognate enzymes involved in xylan biosynthesis in these algae.

## 2. Xylans in Green and Red Algae

### 2.1. Xylans in Charophyte Green Algae

**Figure 2:**
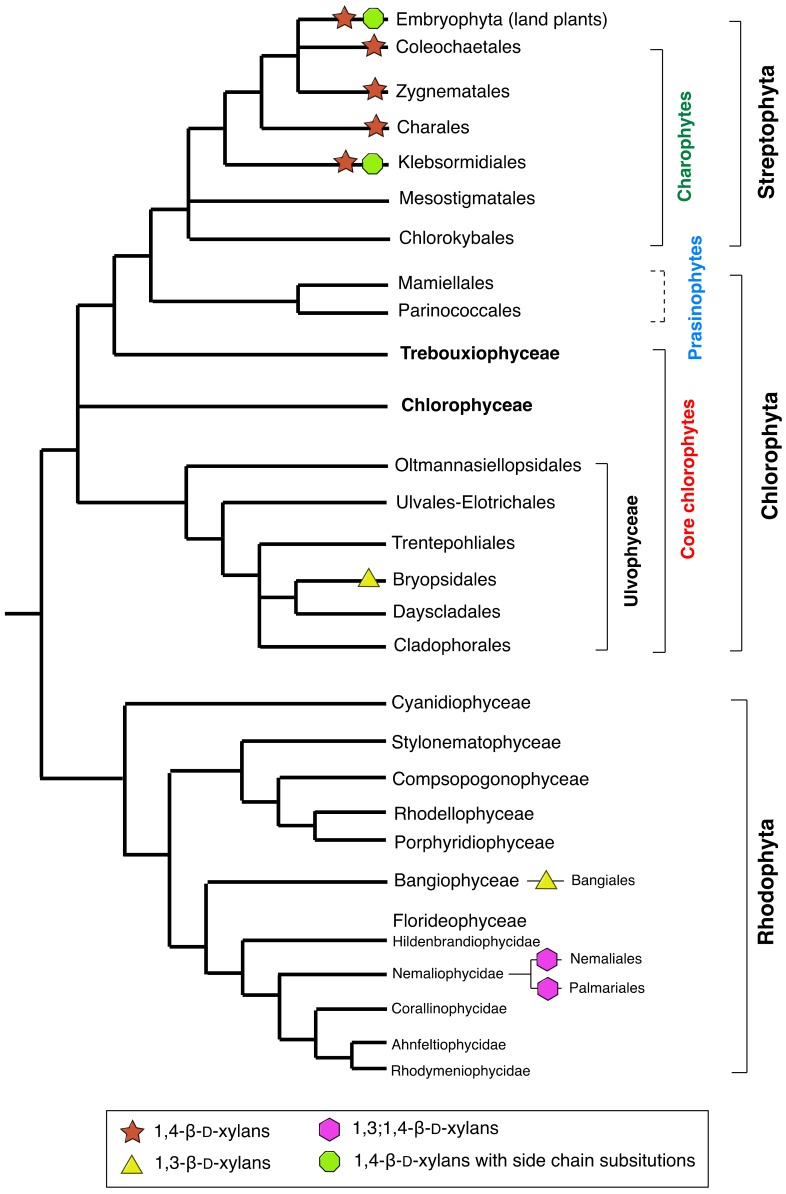
Phylogenies of the red algae [37] and green algae [14] showing the occurrence of xylans. Taxa ending in ‘yta’ are divisions, ‘eae’ are classes, ‘dae’ are subclasses, and ‘les’ are orders.

The phylogenetic tree of green plants is divided into two major clades: the chlorophyte algae (Chlorophyta) and streptophytes (Streptophyta), with the latter comprising the charophyte algae (also known as the charophycean green algae) and land plants [14] (Figure 2). Like land plants, charophytes contain chlorophylls a and b and are recognised as the immediate ancestor of all land plants, occupying a key phylogenetic position for studying land plant origin and evolution [15,16,17]. Charophytes live in a range of terrestrial or freshwater semiaquatic habitats. For example, the alga *Chlorokybus atmophyticus* (Chlorokybales) lives in the soil, and some of the Charales, Klebsormidiales and Zygnematales live in freshwater. The most recent phylogenetic analyses indicate that the Zygnematales, or a clade containing this order and the Coleochaetales, are the closest extant relatives of the land plants [16,17]. A survey has been carried out on the cell wall compositions of 10 species of charophytes representing the advanced orders Charales, Coleochaetales, Zygnematales, Klebsormidiales, and Chlorokybales [18]. Linkage analysis showed that 1,4-linked Xyl*p*, indicative of the presence of 1,4-β-d-xylans, is present, at least in traces, in the cell walls of all the species, with the highest proportions in the cell walls of *Klebsormidium flaccidum* (Klebsormidiales). Furthermore, the presence of 2,4- and/or 3,4-linked Xyl*p* indicate the xylans are probably substituted at *O*-2 and/or *O*-3. The presence of a substituted 1,4-β-d-xylan in the cell walls of *K. flaccidum* has been confirmed in a more recent study [19]. In this study, the xylan was extracted from the cell walls, digested with a pure 1,4-β-d-xylanase and subjected to analysis by PACE (polysaccharide analysis using carbohydrate gel electrophoresis). Treatments with diagnostic xylan α-arabinofuranosidases showed that α-l-Ara*f* substituents are present on the backbone, together with other unidentified substituents. Labelling of the extracted xylan with the monoclonal antibody LM11, which is specific for substituted 1,4-β-d-xylans, was also consistent with the xylan being substituted. In an earlier study of the charophyte alga *Chara corallina*, linkage analysis of the cell walls also showed the presence of 1,4-Xyl*p* linkages and a cell wall extract also labelled with LM11 [20]. These results are all consistent with the charophytes containing 1,4-β-d-heteroxylans similar in structure to those in the cell walls of land plants. The linkage analysis of the cell walls of *K. flaccidum* also showed a significant amount of 1,3-Xyl*p*, a linkage not commonly found in the cell walls of land plants [18]. The linkage could be from 1,3-β-d-xylans, similar to those found in some chlorophyte green algae and some red algae (see Section 2.2 and Section 2.3), but further work is required on this topic.

### 2.2. Xylans in Chlorophyte Green Algae

The chlorophyte algae have very diverse morphologies, ranging from unicellular to complex colonial, thalloid or siphonous forms. Within the class Ulvophyceae (Figure 2), the order Bryopsidales is siphonous [21], and the cell wall microfibrils consist of either 1,3-β-d-xylans or 1,4-β-d-mannans, with cellulose apparently completely absent in most of the genera examined. For example, the microfibrils in the genera *Caulerpa*, *Dichotomosiphon*, *Halimeda*, *Penicillus* and *Udotea* are composed of 1,3-β-d-xylans [22] but in the genera *Codium* and *Derbesia*, they are composed of 1,4-β-d-mannans [23,24,25]. However, in the cell walls of the genus *Bryopsis*, the microfibrils contain both 1,3-β-d-xylans and cellulose [22,26]. The 1,3-β-d-xylans, which appear to be homoxylans, occur in the cell walls as triple helices [27]. Although 1,3-β-d-xylans do not occur in the cell walls of land plants, the structurally related 1,3-β-d-glucans do occur, also as triple helices [28]. A further degree of complexity is added by the finding that in certain species the microfibril composition depends on the stage of the life cycle [29]. For example, although the cell walls of the larger macrothallus phase of *Bryopsis plumosa* (the gametophyte phase for this species) have microfibrils of 1,3-β-d-xylans and cellulose, the cell walls of the microthallus phase (the sporophyte phase) have microfibrils of 1,4-β-d-mannans. On the other hand, the cell walls of the larger macrothallus phase of *Derbesia tenuissima* (the sporophyte phase for this species) have microfibrils of 1,4-β-d-mannans, but the cell walls of the microthallus phase (the gametophyte phase) have microfibrils of 1,3-β-d-xylans. The reasons for these compositional changes between phases and how they are regulated remain unclear.

The sister order to the Bryopsidales, the Dasycladales (Figure 2), which is also siphonous, has genera with cell wall microfibrils composed of 1,4-β-d-mannans and sometimes cellulose, but 1,3-β-d-xylans have apparently not been reported [23,30]. For example, *Acetabularia acetabulum* has cell wall microfibrils with different compositions depending on the region within the plant: microfibrils composed of 1,4-β-d-mannans occur in the vegetative cell walls, but microfibrils composed of cellulose occur in the reproductive cell walls [30]. However, linkage analysis indicated the matrix phase of cell walls in all regions of the plant contain small amounts of 1,4-β-d-xylans that have some substituents, particularly at *O*-2, although the identities of these substituents are unknown [30]. It would be interesting to know whether such xylans, which are similar to those found in charophytes and land plants, are more widespread within the chlorophytes.

### 2.3. Xylans in Red Algae (Rhodophyta)

Most red (rhodophyte) algae are marine and have habitats ranging from coral reefs to deep water, with only 5% found in fresh water. In addition to chlorophylls a and b, red algae have the light-harvesting accessory pigments phycoerythrin and phycocyanin that can reflect red and absorb blue light. Most red algae are multicellular and are often filamentous. With the exception of one order, the Bangiales (within the class Bangiophyceae), the microfibrils in red algae are composed of cellulose, and with the exception of two orders, Nemaliales and Palmariales, within the class Florideophyceae (subclass Nemaliophycidae) (Figure 2), the matrix phase of red algal walls are usually sulphated galactans, including agars and carrageenans [31]. Indeed, the cell wall polysaccharides of red algae have been widely used in red-algal taxonomy [32,33].

Two main types of xylans, both apparently homoxylans, have been reported in red algae: the 1,3-β-d-xylans and the 1,3;1,4-β-d-xylans. The 1,3-β-d-xylans occur as microfibrils in the cell walls of *Porphyra umbilicalis* (Bangiales) [34], where they occur as triple helices as they do in the cell walls of certain genera of the order Bryopsidales in the chlorophytes (Section 2.2). 1,4-β-d-Mannans were also present in this species but in a ‘cuticle’ rather than the cell walls proper. Although not examined in as much detail as *P. umbilicalis*, similar results were also obtained with *Bangia atropurpurea* (= *B. fuscopurpurea*) (Bangiales). Interestingly, as in certain genera of the Bryopsidales, there are differences in the compositions of the microfibrils in the different phases in the life cycle. Thus, although the microfibrils of the larger macrothallus or bangia phase (the gametophyte phase) of *P. umbilicalis* and *B. fuscopurpurea* are composed of 1,3-β-d-xylans, the microfibrils of the microthallus or conchocelis phase (the sporophyte phase) are composed of cellulose [35,36]. Cellulose microfibrils are thus not absent from the cell walls of taxa in the Bangiales.

In contrast to the 1,3-β-d-xylans, the 1,3;1,4-β-d-xylans (or mixed-linkage xylans) occur in the matrix phase of cell walls of red algae in the orders Nemaliales and Palmariales (Figure 2) and can be extracted with hot water [32,38,39,40]. Like the 1,3-β-d-xylans, the 1,3;1,4-β-d-xylan does not occur in the cell walls of land plants. Because *Palmaria* (= *Rhodymenia*) *palmata* (Palmariales) is used as food (dulse) [41], its 1,3;1,4-β-d-xylans, sometimes referred to as rhodymenan, have been examined in great detail. They are linear molecules containing both 1,3- and 1,4-links in the ratio 1:4 [38,42]. An initial study indicated the 1,3-links were regularly distributed [42], but a later study that focused particularly on the 1,3;1,4-β-d-xylans of *Nothogenia erinacea* (Nemaliales) [43], but that also examined the 1,3;1,4-β-d-xylans of *P. palmata*, concluded that the 1,3-links are irregularly distributed in the xylans of both species. The proportions of 1,3- and 1,4-links vary with species and the method of extraction [39,44]. Although water-soluble 1,3;1,4-β-d-xylans appear to be restricted to the orders Nemaliales and Palmariales [32], they have been found in other red algal taxa by using other extraction methods. For example, they have been found in a chlorite extract of *P. umbilicalis* (Bangiales) and in a dilute alkali extract of *Laurencia pinnatifida* (Ceramiales), and may be quite widely distributed.

Small amounts of 1,4-β-d-xylans have also been reported to occur in the walls of *P. palmata* and *Scinaia hatei* (Nemaliales) [45,46]. It is possible that these xylans are parts of 1,3;1,4-β-d-xylans, but they may be independent molecules. If so, it indicates that red algae can also synthesise at least small amounts of 1,4-β-d-xylans. It would be interesting to know whether such xylans occur in red algae outside of the Palmariales and Nemaliales and whether they are homoxylans.

## 3. Biosynthesis of Algal Xylans

Although a considerable amount is known about the biosynthesis of xylans in land plants, information about the biosynthesis of these polysaccharides in green and red algae is confined to the charophyte green algae. Progress in understanding the biosynthesis of land plant heteroxylans was initially confined to genes and cognate proteins involved in the biosynthesis of the various side chains [47,48], including XAT, GUX1-3, GXMT1, and XAX1 (Figure 1). More recently, *Arabidopsis IRX10* (and the highly homologous *IRX10-L*) and two related genes from the eudicotyledon psyllium (*Plantago ovata*) and the moss *Physcomitrella patens* have been heterologously expressed and the proteins produced shown to be xylan 1,4-β-d-xylosyltransferases capable of synthesising long oligosaccharides from UDP-xylose and an acceptor 1,4-β-d-xylooligosaccharide [49,50]. The name xylan synthase-1 (XYS1) has been given to the enzyme [50] (Figure 1).

More recently, a protein in the charophyte alga *Klebsormidium flaccidum* designated *K. flaccidum* xylan synthase-1 (*Kf*XYS1) was identified with 75% amino acid sequence similarity to *Arabidopsis* xylan synthase-1 (*At*XYS1) [19]. After heterologous expression, the protein was shown to have only 1,4-β-d-xylan synthase activity. This indicates that 1,4-β-d-xylan synthases had already evolved in the charophyte algae, and may well be the evolutionary origin of land plant 1,4-β-d-xylans [19]. Other protein (and gene) orthologs to those involved in xylan synthesis in land plants have also been identified, but whether or not they are involved in xylan synthesis in charophytes is unknown. For example, orthologs of IRX9 and IRX14 are found in the Klebsormidiophyceae. In *Arabidopsis*, these proteins have no enzymatic activity and most likely function to anchor IRX10 within Golgi bodies [51]. In addition, although the *K. flaccidum* xylan has α-Ara*f* substituents (see Section 2.1), no orthologs have been found of the GT61 enzymes (XATs) involved in the formation of such side chains in grasses, suggesting that other proteins are used in this organism. Furthermore, nothing is known about the proteins involved in the biosynthesis of the 1,4-β-d-xylans found in the cell walls of the chlorophyte alga *Acetabularia acetabulum* (see Section 2.2) and in some red algae (see Section 2.3).

As far as we are aware, nothing is known about the synthesis of the 1,3-β-d-xylans found in some chlorophyte and red algae, nor the 1,3; 1,4-β-d-xylans found in some red algae or the small amounts of 1,4-β-d-xylans found in both groups. These xylans may have considerable applications in biotechnology and food manufacture, where they will function as dietary fibres. Knowledge about their biosynthesis could guide future manipulation to increase their applied value, as well as shed further light on the biosynthesis and evolution of land plant xylans and how they interact with other cell wall polymers.

## 4. Conclusions

Charophytes contain 1,4-β-d-heteroxylans similar to those that occur in land plants. However, certain taxa of chlorophytes and red algae contain 1,3-β-d-xylans, which are homoxylans. Other taxa of red algae contain 1,3;1,4-β-d-xylans, which are also apparently homoxylans. Small amounts of 1,4-β-d-xylans have also been reported in the cell walls of certain taxa of chlorophytes and red algae, but little is known about their structures. Except for the 1,4-β-d-heteroxylans of the charophytes, nothing is known about the biosynthesis of algal xylans. There is an urgent need for more research leading to a better understanding of their detailed structures and properties, as well as biosynthesis. This information is needed to understand the evolution of land plant xylans, as well as for the possibility of greater utilisation of these xylans in novel foods and in biotechnology.
